# Root Remodeling versus Root Reimplantation in Patients with Bicuspid Aortic Valve and Root Aneurysm

**DOI:** 10.1055/s-0045-1809688

**Published:** 2025-06-12

**Authors:** Fei Xiang, Lin Chen, Eric E. Roselli, Brian Griffin, Milind Desai, Jeevanantham Rajeswaran, Austin Firth, Eugene H. Blackstone, Lars G. Svensson

**Affiliations:** 1 The Aorta Center, Miller Family Heart, Vascular & Thoracic Institute, Cleveland Clinic, Cleveland, Ohio; 2 Department of Thoracic and Cardiovascular Surgery, Miller Family Heart, Vascular & Thoracic Institute, Cleveland Clinic, Cleveland, Ohio; 3 Department of Thoracic and Cardiovascular Surgery, Nanjing First Hospital, Nanjing Medical University, Nanjing, China; 4 Department of Quantitative Health Sciences, Lerner Research Institute, Cleveland Clinic, Cleveland, Ohio; 5 Cardiovascular Outcomes Registries and Research, Miller Family Heart, Vascular & Thoracic Institute, Cleveland Clinic, Cleveland, Ohio, USA

**Keywords:** aortopathy, valve-sparing root replacement, durability, survival

## Abstract

**Background:**

Valve-sparing root replacements are increasingly being performed in patients with bicuspid aortic valve (BAV) and root aneurysm. This study aims to compare the outcomes of patients who underwent root remodeling versus root reimplantation.

**Methods:**

From 2000 to 2022, 206 adults with BAV and root aneurysm (mean age: 47 ± 12 years, 183 [89%] male) underwent root remodeling ( *n* = 32) or reimplantation ( *n* = 174) at Cleveland Clinic. Compared with remodeling, patients in the reimplantation group had more aortic regurgitation (severe 61/174 [35%] vs. 3/32 [9.4%]) and smaller aortic roots (sinus diameter: 4.3 ± 0.56 vs. 4.6 ± 0.47 cm). Operative mortality and morbidity, durability, and time-related mortality were compared.

**Results:**

Patients in both groups underwent additional aortic valve repair (reimplantation vs. remodeling group: figure-of-8 hitch-up stitch 10/174 [5.7%] vs. 14/32 [44%], *p* < 0.001; cusp plication 91/174 [52%] vs. 11/32 [34%], *p* = 0.06). Compared with the remodeling group, aortic clamp time was longer in the reimplantation group (median 136 vs. 76 minutes, *p* < 0.001). Two in-hospital reoperations occurred after remodeling from valve dysfunction. One operative death occurred in each group. At 5 years, severe aortic regurgitation was 16% after remodeling versus 5.0% after reimplantation ( *p* = 0.06), mean gradient 11 versus 10 mm Hg ( *p* = 0.12), aortic valve reoperation 23% versus 6.0% ( *p* = 0.14), and survival 97% versus 95%, respectively ( *p* = 0.71).

**Conclusion:**

Both root remodeling and reimplantation can be safely performed in patients with BAV and root aneurysms with similar midterm outcomes. Although root remodeling is a shorter surgery, less late aortic valve regurgitation and fewer valve reoperations lead us to recommend root reimplantation.

## Introduction

Bicuspid aortic valve (BAV) is commonly associated with proximal aortic aneurysm, which presents as either ascending aorta or root phenotypes. 1 Patients with root phenotype have regurgitant aortic valves and dilated aortic anuli. 2 Preservation or repair of native BAV in this relatively young population by valve-sparing root replacement seems an attractive alternative to a Bentall procedure with its anticoagulation requirement, related thromboembolism, and bleeding complications.

Root reimplantation and root remodeling are currently the two main valve-sparing root replacement approaches. Apart from their widespread application and excellent outcome in the setting of tricuspid aortic valves (TAVs), 3 these approaches to BAV root aneurysms have limited clinical experience, with fewer than 100 cases in most single-center reports. 4 Beckmann and colleagues 5 reported a 20-year survival of 84% and freedom from reoperation of 74% among 50 patients with BAV who underwent root reimplantation. We recently found that the durability of root reimplantation in the BAV setting may be inferior to TAV. 3 Root remodeling is another promising valve-sparing root replacement approach for BAV root aneurysms. Schneider and colleagues 6 reported a 15-year survival of 81% and freedom from reoperation of 78% among 357 patients with BAV root remodeling.

Due to the limited clinical experience so far, there is little evidence regarding whether root reimplantation or remodeling is better for the BAV root phenotype. The choice between the two valve-sparing root replacement approaches now mainly depends on a surgeon's experience and preference. Therefore, in this study, we compared outcomes of patients with BAV root phenotype who underwent root reimplantation versus root remodeling, aiming to provide information to support evidence-based surgical decision-making.

## Patients and Procedures

### Patients

**Fig. 1:**
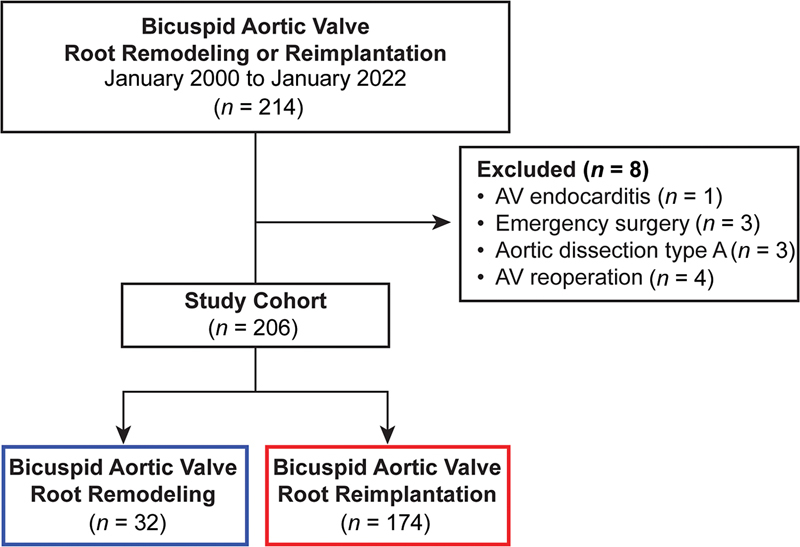
CONSORT-style diagram of patients undergoing bicuspid aortic valve root remodeling or reimplantation. AV, aortic valve.

**Fig. 2:**
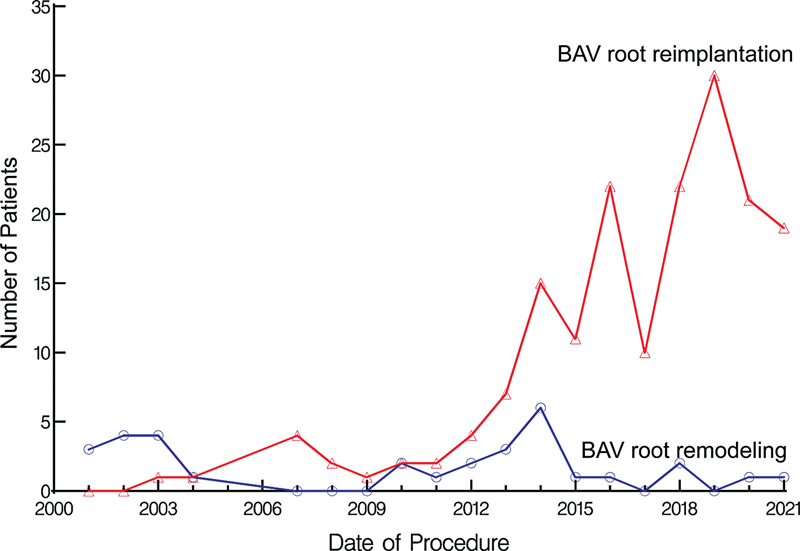
Number of patients undergoing bicuspid aortic valve (BAV) root remodeling (blue line and symbols) or reimplantation (red line and symbols) over the study period. Symbols are a yearly number of cases in each group.

From January 2000 to January 2022, 214 patients underwent valve-sparing root replacement to treat BAV root aneurysm at Cleveland Clinic. After excluding emergency surgery, type A aortic dissection, endocarditis, and aortic valve reoperation, 206 patients were included in the study ( Fig. 1 ), with 11% female and mean age 47 ± 12 years, among whom 32 underwent root remodeling and 174 root reimplantation ( Fig. 1 ). Although the number of root remodeling procedures was nearly constant over time, the number of root reimplantation gradually increased after 2003 ( Fig. 2 ).

Compared with the root remodeling group, patients who underwent root reimplantation had more aortic regurgitation (severe: 61/174 [35%] vs. 3/32 [9.4%]), larger left ventricular end-systolic volume index (26 ± 11 vs. 21 ± 9.1 mL/m ^2^ ), and smaller aortic roots (sinus diameter: 4.3 ± 0.56 vs. 4.6 ± 0.47 cm; Table 1 ).

**Table 1:** Baseline characteristics of patients undergoing bicuspid aortic valve root remodeling or reimplantation

Characteristics	BAV root remodeling ( *n* = 32)		BAV root reimplantation ( *n* = 174)		Standardized mean difference (%) (95% PR b )
	*n* a	Number (%) or mean ± SD	*n* a	Number (%) or mean ± SD	
Demographics:					
Age (years)	32	45 ± 12	174	47 ± 12	−15 (−38, 37)
Female	32	1 (3.1)	174	22 (13)	−36 (−36, 36)
Body surface area (m ^2^ )	32	2.1 ± 0.18	174	2.1 ± 0.25	9.3 (−39, 39)
Aortic regurgitation grade	32	–	174	–	−63 (−36, 36)
None/Trace		15(47)		49(28)	
Mild		8(25)		29(17)	
Moderate		6(19)		35(20)	
Severe		3(9.4)		61(35)	
Aortic root size (diameter):					
Aortic valve anulus (cm)	8	2.7 ± 0.35	63	2.6 ± 0.51	27 (−69, 68)
Aortic sinus (cm)	28	4.6 ± 0.47	159	4.3 ± 0.56	55 (−38, 38)
Sinutubular junction (cm)	22	4.2 ± 0.54	109	3.9 ± 0.59	51 (−52, 49)
Middle ascending aorta (cm)	30	4.5 ± 0.63	157	4.5 ± 0.74	3.3 (−39, 39)
LV morphology and function:					
Ejection fraction (%)	32	57 ± 7.7	174	60 ± 5.8	44 (−37, 39)
End-diastolic volume index (mL/m ^2^ )	31	58 ± 22	157	70 ± 24	−52 (−40, 40)
End-systolic volume index (mL/m ^2^ )	31	21 ± 9.1	156	26 ± 11	−47 (−39, 43)
Mass index (g/m ^2^ )	30	104 ± 35	156	115 ± 39	−24 (−40, 38)
Relative wall thickness (cm)	30	0.44 ± 0.13	157	0.40 ± 0.10	44 (−41, 41)
Other cardiovascular comorbidities:					
Atrial fibrillation or flutter	30	3 (10)	171	15 (8.8)	4.2 (−49, 37)
Heart failure	32	1 (3.1)	173	20 (12)	−33 (−52, 39)
Prior cardiac surgery	32	4 (13)	174	15 (8.6)	13 (−50, 34)
Noncardiac comorbidities:					
Pharmacologically treated diabetes	32	1 (3.1)	174	3 (1.7)	9.1 (−22, 27)
COPD	32	0 (0)	174	6 (3.4)	−27 (−27, 34)
Peripheral artery disease	32	1 (3.1)	174	6 (3.4)	−1.8 (−29, 31)
Hypertension	32	20 (63)	174	104 (60)	5.6 (−40, 38)
History of smoking	31	13 (42)	174	50 (29)	28 (−43, 40)
Dyslipidemia	30	12 (40)	141	55 (39)	2.0 (−41, 40)
Creatinine (mg/dL)	32	1.01 ± 0.14	174	0.99 ± 0.37	6.7 (−29, 29)
Hematocrit (%)	32	43 ± 3.1	174	44 ± 3.6	−31 (−40, 39)
Concomitant procedures:					
Aortic arch repair	32	5 (16)	174	20 (11)	12 (−39, 32)
Coronary artery bypass grafting	32	1 (3.1)	174	7 (4.0)	−4.8 (−31, 27)
Mitral valve repair	32	0 (0)	174	7 (4.0)	−29 (−29, 31)
Procedure for atrial fibrillation	32	1 (3.1)	174	26 (15)	−42 (−42, 37)
Aortic repair techniques:					
Cabrol suture	32	22 (69)	174	25 (14)	132 (−43, 39)
Cusp plication	32	11 (34)	174	91 (52)	−37 (−37, 39)
Figure-of-8 hitch-up stitch	32	14 (44)	174	10 (5.7)	98 (−37, −34)

Abbreviations: BAV, bicuspid aortic valve; COPD, chronic obstructive pulmonary disease; LV, left ventricular; PR, plausible range; SD, standardized deviation.

a Patients with data available.

b Plausible range under the null hypothesis that the population standardized difference is zero based on 1,000 permutations.

### Operative Techniques

For root remodeling, we prefer to use the inclusive technique for left and right coronary reimplanting to achieve better hemostasis. 7 We routinely perform root reimplantation using Svensson's modified approach, 8 placing pledgeted stitches through the aortoventricular junction and reducing the anular size by tying these stitches around a Hegar dilator of customized size (which is typically larger [23–25 mm] as compared to those with a tricuspid valve).

To achieve aortic valve competence, we usually use additional repair techniques. Compared with root remodeling, reimplantation was accompanied more by cusp plication (91/174 [52%] vs. 11/32 [34%]) and supracommissural sutures (47/174 [27%] vs. 1/32 [3.1%]) and less by figure-of-8 hitch-up stitches 9 (10/174 [5.7%] vs. 14/32 [44%]) and Cabrol sutures (25/174 [14%] vs. 22/32 [69%]; Table 2 ). Concomitant cardiac procedures included coronary artery bypass grafting, mitral valve surgery, aortic arch repair, and surgical ablation for atrial fibrillation.

**Table 2:** Operative details and in-hospital outcomes of patients undergoing bicuspid aortic valve root remodeling or reimplantation

Characteristics	BAV root remodeling ( *n* = 32)		BAV root reimplantation ( *n* = 174)		Standardized mean difference (%) (95% PR b )
	*n* a	Number (%) or median [15th, 85th percentile]	*n* a	Number (%) or Median [15th, 85th percentile]	
**Operative details:**					
**Aortic repair techniques:**					
Cabrol suture	32	22 (69)	174	25 (14)	132 (−43, 39)
Cusp plication	32	11 (34)	174	91 (52)	−37 (−37, 39)
Figure-of-8 hitch-up stitch	32	14 (44)	174	10 (5.7)	98 (−37, −34)
**Concomitant procedures:**					
Aortic arch repair	32	5 (16)	174	20 (11)	12 (−39, 32)
Coronary artery bypass grafting	32	1 (3.1)	174	7 (4.0)	−4.8 (−31, 27)
Mitral valve repair	32	0 (0)	174	7 (4.0)	−29 (−29, 31)
Procedure for atrial fibrillation	32	1 (3.1)	174	26 (15)	−42 (−42, 37)
**In-hospital outcomes:**					*p* -Value
Operative death	32	1 (3.1)	174	1 (0.57)	0.29
Permanent stroke	32	1 (3.1)	174	0 (0)	0.16
Reoperation for valve dysfunction	32	2 (6.3)	174	0 (0)	0.02
Reoperation for bleeding or tamponade	32	0 (0)	174	5 (2.9)	>0.99
Any blood product transfusion	32	14 (44)	173	78 (45)	0.89
New requirement for dialysis	27	0 (0)	171	2 (1.2)	>0.99
Prolonged ventilation	29	0 (0)	174	2 (1.1)	>0.99
New postoperative atrial fibrillation	27	4 (15)	156	34 (22)	0.61
AR severity at discharge	21		142		0.04
None		17(81)		130 (92)	
Mild		2 (9.5)		11 (7.8)	
Moderate		1 (4.8)		1 (0.70)	
Severe		1 (4.8)		0 (0)	
ICU length of stay (hours)	32	32 [22, 73]	172	39 [21, 70]	0.69
Operative length of stay (days)	32	6 [5, 6]	174	6 [5, 8]	0.56

Abbreviations: AR, aortic regurgitation; BAV, bicuspid aortic valve; ICU, intensive care unit; PR, plausible range.

a Patients with data available.

b Plausible range under the null hypothesis that the population standardized difference is zero based on 1,000 permutations.

### Data

Preoperative patient characteristics and operative details were collected prospectively for quality reporting by independent registry nurses and entered into the cardiovascular information registry. Transthoracic echocardiographic data were measured and entered into the echocardiography database by clinical echosonographers. Other Cleveland Clinic electronic medical record databases were also queried. All data used for this study were approved for use in research by the Cleveland Clinic Institutional Review Board, with patient consent waived (IRB #22-671, approved July 11, 2022).

### Endpoints

#### Operative Mortality and Morbidity

Operative mortality included in-hospital deaths and any death occurring after hospital discharge but within 30 days after surgery. In-hospital morbidity was defined according to the Society of Thoracic Surgeons National Database.

#### Longitudinal Echocardiographic Outcomes

Aortic valve regurgitation grade (none, mild, moderate, or severe), mean gradient, and left ventricular mass index were assessed on serial postoperative echocardiograms. A total of 123 echocardiograms were available for 75% of patients (24/32) in the remodeling group, and 531 echocardiograms were available for 94% of patients (164/174) in the reimplantation group ( Supplementary Fig. S1 [available in the online version]). All longitudinal measurements were censored at reoperation.

#### Time-related Aortic Valve Reoperation and Mortality

To assess reoperations on the aortic valve and vital status, patients underwent follow-up systematically at 2 and 5 years and 5-year intervals thereafter, via mailed questionnaires or telephone contact with the patient or family member. Systematic follow-up for vital status was supplemented with Social Security Death Master File data (to 2011) and Ohio State Death Registry data. The median follow-up duration for reoperation on the aortic valve in the remodeling group was 2.0 years, with 25% of patients undergoing follow-up of more than 5.3 years and 10% for more than 15 years. In the reimplantation group, the median follow-up duration was 2.0 years, with 25% of patients undergoing follow-up for more than 5.0 years and 10% for more than 15 years. The median follow-up duration for vital status in the remodeling group was 5.1 years, with 25% undergoing follow-up more than 13 years and 10% more than 18 years. In the reimplantation group, the median follow-up duration was 3.0 years, with 25% followed more than 5.5 years and 10% more than 8.3 years.

### Statistical Analysis

Statistical analyses were performed using SAS version 9.4 (SAS Institute, Cary, NC) and R software version 4.0.3 (R Foundation for Statistical Computing, Vienna, Austria). Continuous variables are summarized as mean ± standard deviation or as equivalent 15th, 50th (median), and 85th percentiles when the distribution of values was skewed. Categorical data are summarized by frequencies and percentages. Continuous outcomes were compared using the Wilcoxon rank-sum test and categorical outcomes using the chi-square test or Fisher's exact test, if appropriate. Confidence intervals for longitudinal estimates used a bootstrap percentile method to obtain 68% confidence bands (equivalent to ±1 standard error) and the delta method for time-related events. A type I error of 0.05 was used to assess statistical significance. For baseline characteristics, standardized mean differences (SMDs) were used for comparison. An absolute value of SMD equal to or less than 10% is usually interpreted as an acceptable difference. 10 However, when the sample size is smaller in the overall cohort or one group, as in this study, there may be larger variability in SMD that could yield estimated SMD values greater than 10%. To assess if the true underlying SMD is zero, we estimated a 95% plausible range using the empirical distribution of SMD from 1,000 permutations under the null hypothesis that population SMD is zero. 11 Any estimated SMD not falling within the interval was considered imbalanced.

#### Echocardiographic Longitudinal Analysis

To assess the temporal trend of individual grades of postoperative aortic regurgitation (ordinal longitudinal data), follow-up transthoracic echocardiograms were analyzed for the pattern of change across time using a nonlinear, multiphase, mixed-effects, cumulative logistic regression model. 12 The model resolved the number of time phases to form a temporal decomposition model and estimate its shaping parameters. The prevalence of each aortic regurgitation grade over time was estimated by averaging the patient-specific profiles. A multiphase, nonlinear, mixed-effects regression model for continuous longitudinal data was used to estimate the temporal trend of postoperative mean gradient and left ventricular mass index. 13

#### Time-related Analysis

Survival was estimated nonparametrically by the Kaplan–Meier method. Freedom from aortic valve reoperation was similarly estimated but with patients censored for the competing risk of death.

## Results

### Operative Mortality and Morbidity

Aortic clamp time was longer for root reimplantation compared with root remodeling (median 136 [15th and 85th percentiles of 4 and 162, respectively] vs. 76 [15th and 85th percentiles of 56 and 106, respectively] minutes, *p* < 0.001), as was cardiopulmonary bypass time (median 153 [15th and 85th percentiles of 121 and 192, respectively] vs. 97 [15th and 85th percentiles of 67 and 136, respectively] minutes, *p* < 0.001). One operative death occurred in each group ( Table 2 ). The remodeling group had more in-hospital reoperations due to valve dysfunction (2/32) than the reimplantation group (0/174). Occurrence of other in-hospital outcomes was similar between groups ( Table 2 ), including blood transfusions, reoperation for bleeding, and intensive care unit (median 39 [15th and 85th percentiles of 21 and 70, respectively] vs. 32 [15th and 85th percentiles of 22 and 73, respectively] hours, *p* = 0.69) and postoperative (median 6 [15th and 85th percentiles of 5 and 8, respectively] vs. 6 [15th and 85th percentiles of 5 and 6, respectively] days, *p* = 0.56) lengths of stay. Prevalence of moderate or more aortic valve regurgitation at discharge was 2/21 (9.5%) in the remodeling group and 1/142 (0.70%) in the reimplantation group ( *p* = 0.04; Table 2 ).

### Longitudinal Echocardiographic Trends

**Fig. 3:**
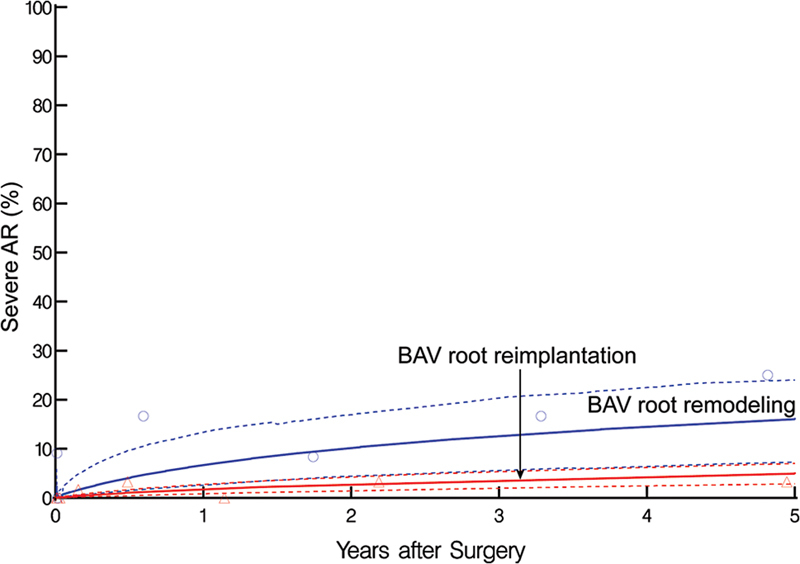
Postoperative prevalence of severe aortic regurgitation (AR) in patients undergoing bicuspid aortic valve (BAV) root remodeling (blue lines and symbols) or reimplantation (red lines and symbols). Solid lines represent longitudinal trends enclosed within a dashed 68% confidence band, and symbols represent data grouped (without regard to repeated measurements) within time frames to provide crude verification of model fit.

**Fig. 4:**
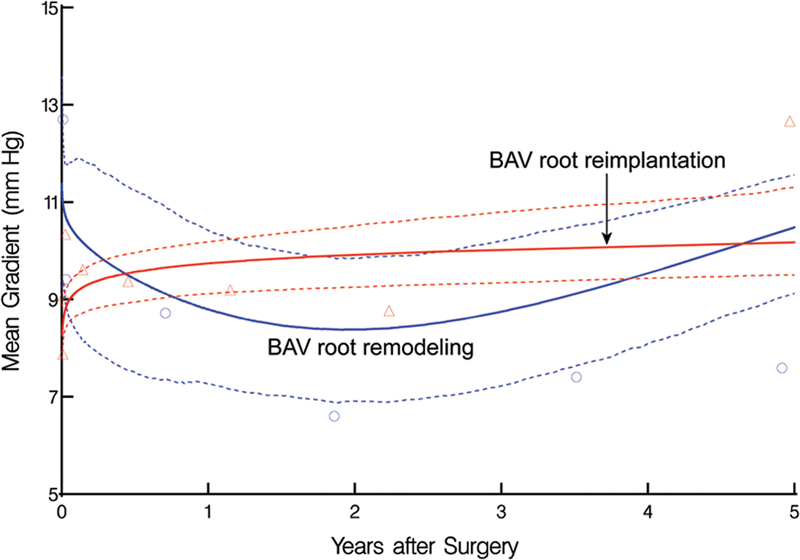
Postoperative trend of the mean gradient in patients undergoing bicuspid aortic valve (BAV) root remodeling (blue lines and symbols) or reimplantation (red lines and symbols). The format is as in Fig. 3.

At 1 year postoperatively, prevalence of severe aortic regurgitation was 6.7% in the remodeling group versus 1.8% in the reimplantation group. At 5 years, these proportions increased to 16% versus 5.0%, respectively ( *p* = 0.06; Fig. 3 ). No significant difference was found in aortic valve mean gradient between the groups after index surgery (remodeling vs. reimplantation: 8.8 vs. 9.7 mm Hg at 1 year, and 11 vs. 10 mm Hg at 5 years, *p* = 0.12; Fig. 4 ). At 1 year postoperatively, mean left ventricular mass index was higher in the remodeling group versus the reimplantation group (115 vs. 101 g/m ^2^ , *p* = 0.05). However, no significant difference was found at 5 years postoperatively (105 vs. 100 g/m ^2^ , *p* = 0.52; Supplementary Fig. S2 [available in the online version]).

### Time-related Reoperation and Mortality

**Fig. 5:**
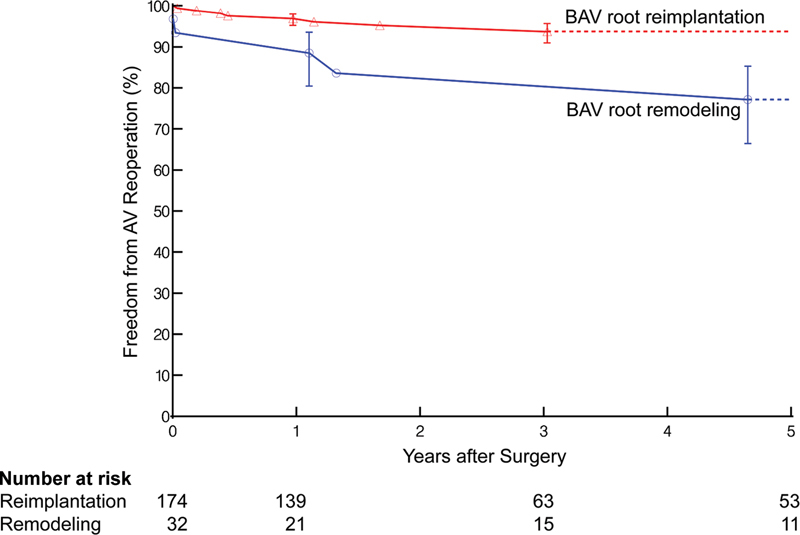
Freedom from aortic valve (AV) reoperation after bicuspid aortic valve (BAV) root remodeling (blue lines and symbols) or reimplantation (red lines and symbols). Each symbol represents a Kaplan–Meier estimate of the event, and vertical bars are 68% confidence limits equivalent to ±1 standard error. Numbers below the horizontal axis are patients remaining at risk.

There were 6 aortic valve reoperations after root remodeling and 10 after root reimplantation. Freedom from aortic valve reoperation was lower after remodeling than reimplantation (93 vs. 99% at 30 days, 93 vs. 97% at 1 year, and 77 vs. 94% at 5 years [ *p* = 0.14]; Fig. 5 ). Reasons for reoperation after root remodeling included cusp prolapse, natural progression, and tearing ( Supplementary Table S1 [available in the online version]). After root reimplantation, these included cusp perforation, infection, disease progression, and tearing.

**Fig. 6:**
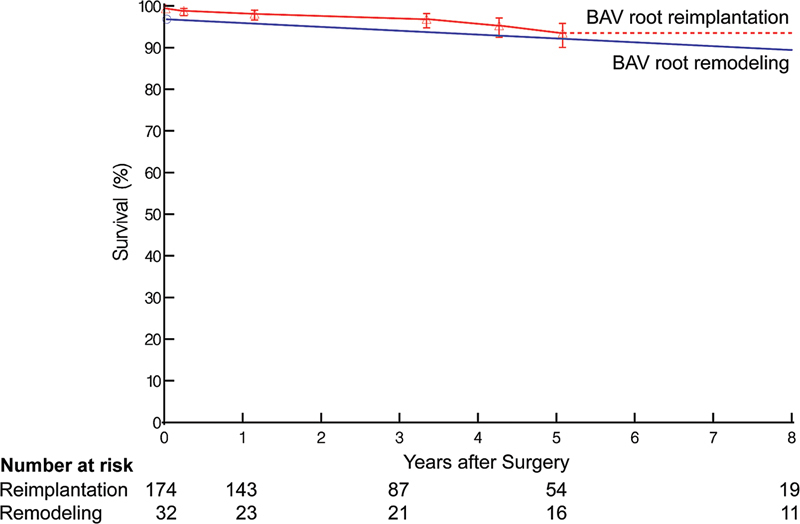
Survival after bicuspid aortic valve (BAV) root remodeling (blue lines and symbols) or reimplantation (red lines and symbols). The format is as in Fig. 5 .

Nine deaths occurred during follow-up: three in the remodeling group and six in the reimplantation group. No significant difference was found in risk of death between the two groups ( *p* = 0.71; Fig. 6 ). Survival estimates after root remodeling versus reimplantation were 96.8 versus 98.8% at 1 year, 96.8 versus 95.3% at 5 years, and 96.8 versus 93.5% at 8 years ( *p* = 0.71; Fig. 6 ).

## Discussion

### Principal Findings

This comparative study showed that root reimplantation and root remodeling could be performed safely and had good outcomes in patients with BAV and a root aneurysm. However, root implantation has better durability, including fewer aortic valve reoperations and less severe aortic valve regurgitation.

### Findings in Context

Aortic root anatomy and repair logic differ between bicuspid and tricuspid valve pathology. A regurgitant TAV root reimplantation effectively downsizes the aortic anulus, which not only increases coaptation height but also prevents later dilatation of the aortic anulus by securing the aortoventricular junction. However, the anatomy of a BAV root has more variations, including cusp fusion, commissural orientation, and location of coronary ostia, so we believe that valve-sparing aortic root replacement is more challenging in a BAV root than a TAV. It has also been pointed out that root reimplantation in a BAV root may not be as effective as in a TAV root because reimplantation of a BAV into a tube graft often reduces intercommissural distance and induces cusp prolapse. 7 By contrast, a large tube graft for root remodeling puts tension on the cusps longitudinally, which helps secure the competence of bicuspid aortic cusps. Concerns about root remodeling include bleeding risk from the long anastomosis line and lack of support for the aortic anulus. 7

In this study, we showed that both root remodeling and reimplantation can be safely performed to treat root aneurysms and preserve or repair regurgitant BAVs. Likely because it did not need deep dissection around the aortic root or placement of aortoventricular junction stitches, root remodeling required less aortic clamp and on-pump time. In addition, root remodeling did not have a higher risk of intraoperative and postoperative bleeding complications compared with root reimplantation, which may be attributed to Svensson's inclusive technique. Nevertheless, more patients in the remodeling group required additional commissural elevation and anuloplasty by figure-of-8 hitch-up stitches and Cabrol sutures, and still, there were two early repair failures (2/32) after root remodeling. Conversely, we did not see any in-hospital recurrence of moderate or severe aortic regurgitation after root reimplantation.

Despite the similarly good operative results in both groups, root remodeling may not be as durable as reimplantation. During follow-up, we found that patients who underwent root remodeling were more likely to have a recurrence of clinically important aortic regurgitation or receive an aortic valve reoperation than those who underwent root reimplantation despite having less severe aortic regurgitation preoperatively. As our preoperative patient characteristics show, a majority of patients in this cohort had dilated aortic anuli with a mean diameter exceeding 2.6 cm. We assume that dilated aortic anuli may be associated with inferior long-term durability since root remodeling was not able to effectively secure the aortic anulus even with Cabrol sutures. This finding reinforces the belief that a complete anuloplasty provided by the reimplantation strategy is preferred. Despite consistent anulus reduction, no significant increase in aortic valve gradient was found within 5 years after remodeling or reimplantation. Longer follow-up is needed to determine the long-term progression of BAV pathology after valve-sparing root replacement and to guide refinements in patient selection and techniques.

### Limitations

This is an observational clinical study at a single institution. Due to the limited sample size in the remodeling group, we could not perform the propensity-score matching to minimize confounding bias. In addition, our echocardiographic follow-up data were available only for patients routinely followed at our institution. Longer follow-up is needed to assess longer-term outcomes.

## Conclusion

Both root remodeling and reimplantation can be safely performed in patients with BAV and root aneurysms with similarly good midterm outcomes. Although root remodeling is a shorter operation than reimplantation, less late aortic valve regurgitation and fewer valve reoperations lead us to recommend reimplantation. A better understanding of BAV root anatomy and more technological innovation is needed to improve its long-term outcome.
