# Supplementary material for: Root Remodeling versus Root Reimplantation in Patients with Bicuspid Aortic Valve and Root Aneurysm
Source: Aorta (Stamford). 2025 Jun 12;13(1):1–8. doi: 10.1055/s-0045-1809688 (PMC12202024; doi:10.1055/s-0045-1809688)
Supplement: Supplementary file 1 — Supplementary Material [file 10-1055-s-0045-1809688-s230026.pdf]

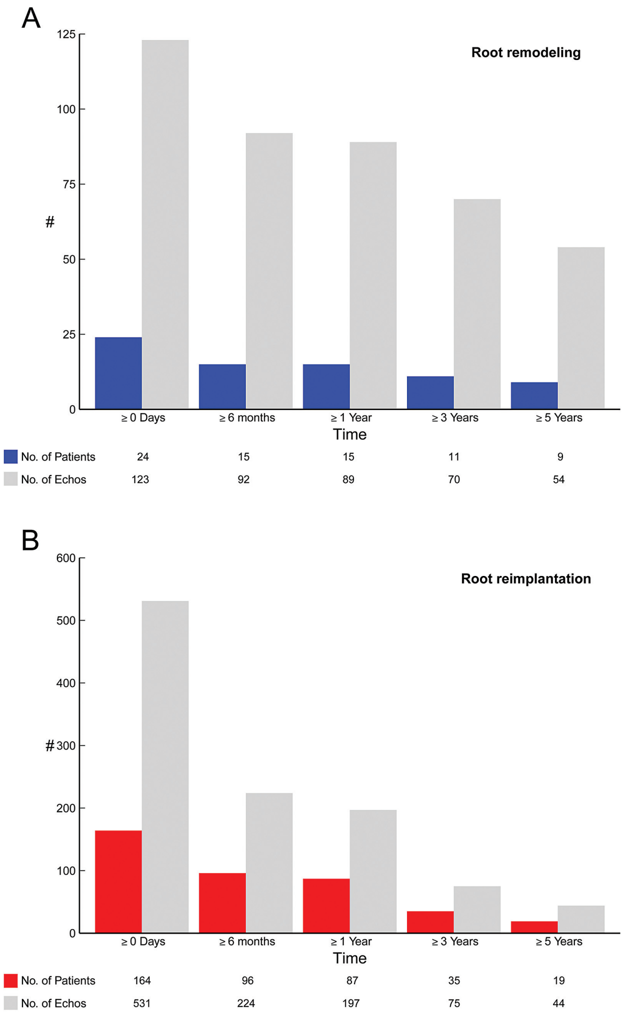

Supplementary Fig. S1 Echocardiographic follow-up across the study period shows the number of patients with echocardiograms and the number of follow-up echos available at and beyond designated time points. (**A**) Bicuspid aortic valve root remodeling. One hundred and twenty-three echocardiograms were evaluated for 24 patients. (**B**) Bicuspid aortic valve root reimplantation. Five hundred and thirty-one echocardiograms were evaluated for 164 patients.

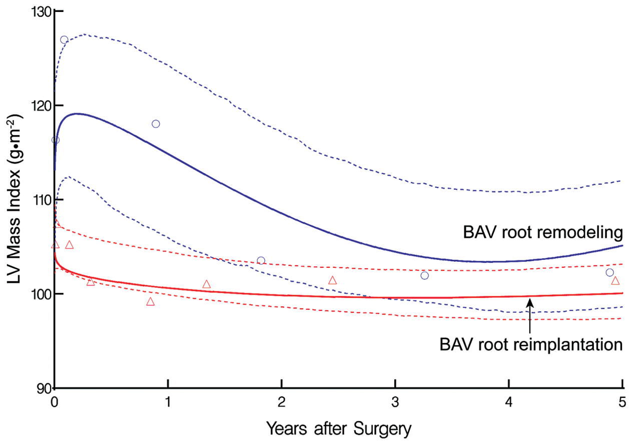

Supplementary Fig. S2 Postoperative trend of left ventricular (LV) mass index in patients undergoing bicuspid aortic valve (BAV) root remodeling (blue lines and symbols) or reimplantation (red lines and symbols). The format is as in Fig. 3.

Supplementary Table S1 Reasons for aortic valve reoperation after aortic bicuspid valve root remodeling or reimplantation

Reasons	BAV root remodeling (number of patients)	BAV root reimplantation (number of patients)
Cusp perforation	0	2
Cusp prolapse	2	0
Cusp tear	2	2
Natural progression	2	1
Infectious endocarditis	0	1
Unknown	0	4
Total	6	10

Abbreviation: BAV, bicuspid aortic valve.

 © 2025. The Author(s).
